# The alterations of oral, airway and intestine microbiota in chronic obstructive pulmonary disease: a systematic review and meta-analysis

**DOI:** 10.3389/fimmu.2024.1407439

**Published:** 2024-05-08

**Authors:** Ziwei Kou, Kai Liu, Zhengtong Qiao, Yaoyao Wang, Yanmiao Li, Yinan Li, Xinjuan Yu, Wei Han

**Affiliations:** ^1^ Department of Medicine, Qingdao University, Qingdao, China; ^2^ Department of Rehabilitation Medicine, Qingdao Municipal Hospital, University of Health and Rehabilitation Sciences, Qingdao, China; ^3^ School of Rehabilitation Medical, Binzhou Medical University, Yantai, China; ^4^ Clinical Research Center, Qingdao Key Laboratory of Common Diseases, Qingdao Municipal Hospital, University of Health and Rehabilitation Sciences, Qingdao, China; ^5^ Department of Respiratory and Critical Medicine, Qingdao Key Laboratory of Common Diseases, Qingdao Municipal Hospital, University of Health and Rehabilitation Sciences, Qingdao, China

**Keywords:** human oral microbiome, human airway microbiome, human intestine microbiome, alpha-diversity, beta-diversity, relative abundance, chronic obstructive respiratory disease, meta-analysis

## Abstract

**Background:**

Increasing evidence indicates the microbial ecology of chronic obstructive pulmonary disease (COPD) is intricately associated with the disease’s status and severity, and distinct microbial ecological variations exist between COPD and healthy control (HC). This systematic review and meta-analysis aimed to summarize microbial diversity indices and taxa relative abundance of oral, airway, and intestine microbiota of different stages of COPD and HC to make comparisons.

**Methods:**

A comprehensive systematic literature search was conducted in PubMed, Embase, the Web of Science, and the Cochrane Library databases to identify relevant English articles on the oral, airway, and intestine microbiota in COPD published between 2003 and 8 May 2023. Information on microbial diversity indices and taxa relative abundance of oral, airway, and intestine microbiota was collected for comparison between different stages of COPD and HC.

**Results:**

A total of 20 studies were included in this review, involving a total of 337 HC participants, 511 COPD patients, and 154 AECOPD patients. We observed that no significant differences in alpha diversity between the participant groups, but beta diversity was significantly different in half of the included studies. Compared to HC, *Prevotella*, *Streptococcus*, *Actinomyces*, and *Veillonella* of oral microbiota in SCOPD were reduced at the genus level. Most studies supported that *Haemophilus*, *Lactobacillus*, and *Pseudomonas* were increased, but *Veillonella*, *Prevotella*, *Actinomyces*, *Porphyromonas*, and *Atopobium* were decreased at the genus level in the airway microbiota of SCOPD. However, the abundance of *Haemophilus*, *Lactobacillus* and *Pseudomonas* genera exhibited an increase, whereas *Actinomyces* and *Porphyromonas* showed a decrease in the airway microbiota of AECOPD compared to HC. And *Lachnospira* of intestine microbiota in SCOPD was reduced at the genus level.

**Conclusion:**

The majority of published research findings supported that COPD exhibited decreased alpha diversity compared to HC. However, our meta-analysis does not confirm it. In order to further investigate the characteristics and mechanisms of microbiome in the oral-airway- intestine axis of COPD patients, larger-scale and more rigorous studies are needed.

**Systematic review registration:**

PROSPERO (https://www.crd.york.ac.uk/prospero/), identifier CRD42023418726.

## 1 Introduction

Chronic obstructive pulmonary disease (COPD) is a prevalent chronic lung condition characterized by persistent airway inflammation and irreversible airway remodeling. The increasing global aging population has led to the recognition of COPD as a significant cause of mortality worldwide, particularly in low- and middle-income countries, thereby posing a substantial public health concern (1). In China, the number of deaths attributed to COPD exceeded 9 million in 2013, with over 99 million adults aged 20 and above diagnosed with COPD based on spirometry measurements in 2015. The high prevalence and mortality rate of COPD impose considerable burdens on the economy, society, and healthcare resources (2). Although environmental factors, genetic susceptibility, lung inflammation, and oxidative stress are widely acknowledged mechanisms contributing to the development of COPD; they do not fully elucidate its occurrence or provide effective interventions. Consequently, research focus has gradually shifted from molecular mechanisms towards exploring the host’s microbial ecological environment.

Based on estimations, the human body harbors a microbial community comprising approximately 100 trillion cells, surpassing the number of host cells. Furthermore, these complex communities of microbes that include bacteria, fungi, protozoa and viruses exhibit a greater diversity of unique genes in comparison to the host genome and play an essential role in regulating various physiological functions (3). Mounting evidence suggested that microbiota actively participate in regulating inflammation signaling pathways, epigenetic modifications, immune responses, and generation of microbiota metabolites to influence the development of host diseases (4). In recent years, advancements in high-throughput sequencing technology had expanded the scope of microbial research. This had progressively unveiled bidirectional connections between oral-lung and intestine-lung microbiota with microbes acting as intermediaries shuttling between these three regions. Microbial alterations are associated with pathological changes linked to various diseases. Current studies indicated differences in the microbial composition of the oral cavity, airways, and intestine between COPD and HC, which could be influenced by disease status and severity (5, 6).

To date, the majority of research findings had supported the concept that HC exhibited higher microbial alpha diversity compared to COPD. However, conflicting results arise from certain studies suggesting increased microbial diversity in diseased states. Although some discrepancies could be attributed to limited sample sizes, variations in sampling techniques, and different sequencing platforms, these factors did not fully account for the observed heterogeneity in these studies. The objective of this meta-analysis is to compare microbial diversity between COPD and HC across three distinct host sites: oral cavity, airway, and intestine. By analyzing available data on differential microbial communities among hosts with varying disease statuses, this study aims to identify specific bacterial biomarkers for diagnosing COPD and enhance our understanding of its pathogenesis. Ultimately, it provides a theoretical basis for diagnosing, intervening in, and treating microbiota-related diseases.

## 2 Materials and methods

This systematic review had been registered in PROSPERO (as CRD42023418726) and searches were conducted in accordance with the updated 2020 Preferred Reporting Items for Systematic Reviews and Meta-Analyses (PRISMA) statement and checklist (7).

### 2.1 Data sources and search strategy

A systematic literature search was conducted in four databases: Pubmed, Web of Science, Embase, and The Cochrane Library. This study exclusively focused on original human research articles published in English between 2003 and 8 May 2023. Relevant literature pertaining to the bacterial microbiota of COPD was limited to three different cavities: oral cavity, airway, and intestine. The search strategy was formed by medical subject headings (MeSH) words and their corresponding free words to minimize the risk of missing relevant literature. The MeSH words used in this article are ‘Microbiota’ [Mesh] and ‘Pulmonary Disease, Chronic Obstructive’ [Mesh]. The search strings are provided in **Supplementary Table 1**.

To ensure the comprehensiveness of the literature search, we conducted a secondary search using the “backward snowballing” method to identify relevant articles that were not captured through equation recognition from the reference list of included studies. A total of four additional articles were discovered during the secondary search.

### 2.2 Eligibility criteria

The titles and abstracts were independently screened by two investigators. Any discrepancies between the two researchers were resolved through consensus with a third researcher. To ensure consistent screening criteria, all three investigators underwent standardized training prior to conducting the formal literature screening. The inclusion criteria for studies are as follows (1): the study population comprises individuals aged over 18 years with COPD and HC (2); the study design is an observational case-control or cross-sectional study published in English (3); the article describes microbial characteristics in either oral cavity, airway, or intestine sites of COPD (4); metagenomic sequencing or 16S rRNA sequencing analysis was performed (5); reports on microbial diversity indices are provided in either the article or its **Supplementary Materials** (including tables and figures), with specific numerical values extractable from relevant charts; and (6) there is at least one control group consisting of HC or stable disease patients, as well as at least one case group comprising stable disease patients or exacerbation patients.

Studies meeting any of the following criteria will be excluded (1): studies conducted on non-human populations or individuals under 18 years old (2); studies lacked a clear representation of microbial diversity index or specific data extraction from microbial diversity charts (3); studies without a control group for comparison; and (4) article types such as abstracts, case reports, expert opinions, reviews, letters, or editorials.

All studies were selected based on the aforementioned eligibility criteria, with a primary focus on assessing bacterial alpha-diversity in different cavities of both COPD and HC. This encompassed evaluating metrics such as the Chao1 index, Richness, Shannon index, and Simpson index. Initially, articles were screened based on their titles and abstracts, followed by a thorough examination of the full text to identify those that ultimately met the inclusion criteria. The final selection was reached through consensus among all authors.

### 2.3 Data extraction and synthesis

The necessary data from the included studies were extracted by two researchers and recorded in a self-designed Excel spreadsheet. The extracted data encompassed the following variables (1): study information (title, first author’s name, publication year, journal of publication, study design type, sample type) (2); population characteristics (sample size, different disease states of COPD, age) (3); community-level measurements of microbial composition in different cavities of the host body (oral cavity, airway, and intestine), with a primary focus on α-diversity followed by β-diversity. Taxonomic discoveries at the phylum and genus levels were also included (bacterial relative abundance) (4); sequencing information (NGS sequencing method, amplification region of 16S rRNA). Quantitative parameters for Alpha-diversity were obtained using Get Data Graph Digitizer software when necessary to extract specific data from charts.

### 2.4 Quality assessment and risk of bias

**Figure 1:**
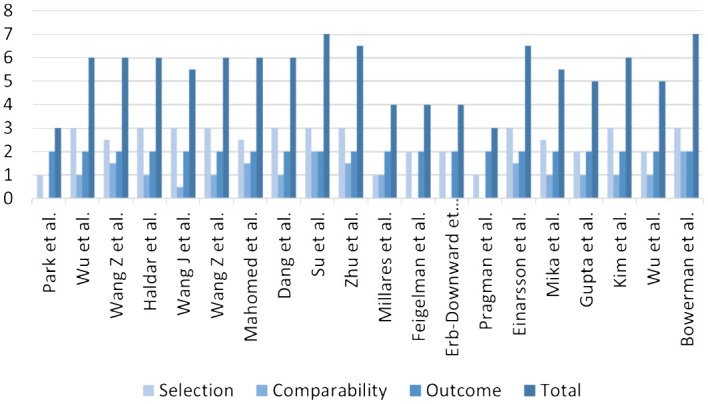
Quality score of included articles calculated using the NOS.

The included studies were independently assessed by the two investigators using the Newcastle-Ottawa Scale (NOS), which was a validated tool for evaluating bias risk in non-randomized studies. The NOS assigned scores based on three key aspects of the included studies: selection, comparability, and outcome. In this assessment process, the selection section had a maximum score of 4 points, comparability had 2 points, and outcome had 3 points, resulting in a total possible score of 9 points. Studies achieving scores between 7-9 were considered high-quality research, while those scoring between 4-6 were classified as moderate-quality research. Studies with scores below 4 were regarded as low-quality research. The specific scores of each article according to the NOS are shown in **Figure 1**; **Supplementary Table 2**.

### 2.5 Statistical analysis

Standardized mean differences (SMDs) were employed to summarize and analyze the variations in microbial diversity between observation groups and control groups for different study subjects using Revman 5.4 software. The differences were estimated with a 95% confidence interval (CI). For quantitative data, means (M) and standard deviations (SD) were used for description, while studies reporting median and interquartile range provided estimates of M and SD through conversion formulas, where SD was calculated as the interquartile range divided by 1.35. Regarding qualitative data such as relative abundance of microbial communities, results from multiple studies were pooled together. Forest plots were generated using random effects models to visualize the disparities in microbial community structures among samples from different study subjects. Sensitivity analysis was performed using Begg’s test and Egger’s test in Stata 12.0 software.

## 3 Results

### 3.1 Search results and study eligibility

**Figure 2:**
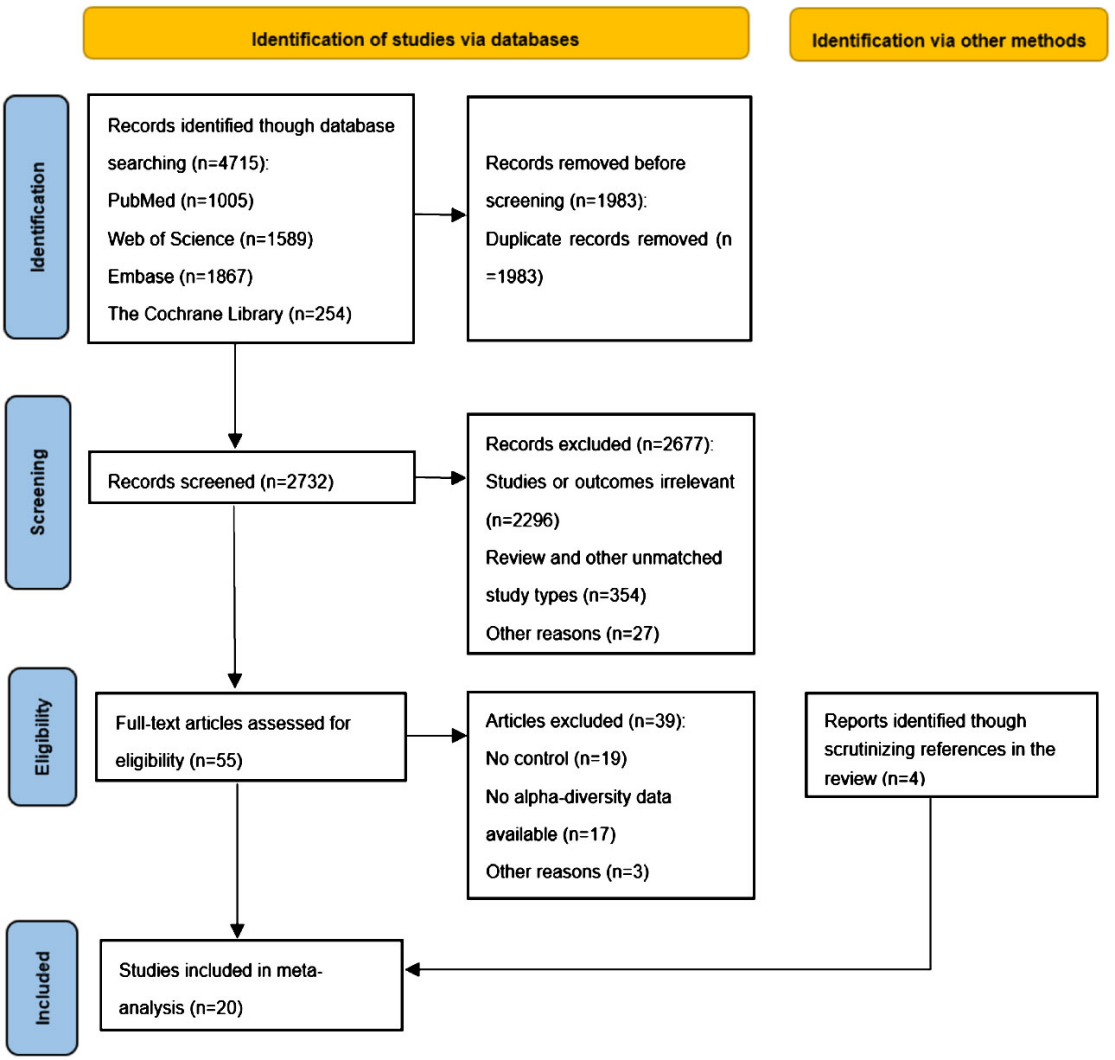
PRISMA flow diagram of selected studies for inclusion.

We retrieved a total of 4,715 articles from four databases (Pubmed, n=1005; Web of Science, n=1589; Embase, n=1867; The Cochrane Library, n=254). Using EndNote software and manual screening to eliminate duplicate studies, we identified 1,983 unique articles. Subsequently, we screened the remaining 2,732 articles based on their titles and abstracts. During this stage, 2,677 articles were excluded primarily due to their lack of relevance to our study or its outcomes (n=2296) and inconsistency with the study type (n=354). After this round of screening, we conducted a full-text review of 55 studies and determined that 16 studies met our inclusion criteria for systematic review. Additionally, employing a “ backward snowballing “ approach, we included an additional four studies. Ultimately, a total of 20 studies were incorporated into our literature review. **Figure 2** provides an overview of the entire process involved in study selection.

The characteristics of the studies included in the meta-analysis were presented in **Table 1**. Among them, two studies focused on investigating the oral microbiota of COPD, utilizing oropharyngeal swabs and periodontal plaques as sample types. In terms of examining the respiratory microbiota of COPD, sixteen studies were identified, employing sputum (n=10), bronchoalveolar lavage fluid (BALF) (n=5), and lung tissue (n=1) as sample types. Regarding the investigation of intestinal microbiota in COPD, two studies utilized fecal samples. Study design-wise, two studies adopted a cross-sectional approach while the remaining seventeen studies were case-control studies. Additionally, there was heterogeneity observed in bacterial sequencing methods employed across these studies; specifically, eleven studies utilized Illumina^©^ systems (primarily MiSeq platform) for sequencing which had the highest representation. Furthermore, six studies targeted V3-V4 regions which was found to be most commonly used for amplification of 16S rRNA gene sequences.

**Table 1:** Characteristics of each study included in the meta-analysis.

Study	Site	Design	Type of sample	NGS sequencing	Region	n_0_	n_1_	n_2_
Park et al. (8)	Oral	Case-control	Oropharyngeal swab	454 pyrosequencing	V1-V3	12	17	–
Wu et al. (9)	Oral	Case-control	Periodontal plaque	Illumina	V4-V5	25	25	–
Wang et al. (10)	Airway	Case-control	Sputum	Illumina	V4	16	43	–
Haldar et al. (11)	Airway	Case-control	Sputum	Illumina	V4	73	60	–
Wang et al. (12)	Airway	Case-control	Sputum	Illumina	V3-V4	10	4	36
Wang et al. (13)	Airway	Case-control	Sputum	PacBio/Illumina	V1-V9	27	98	–
Mahomed et al. (14)	Airway	Case-control	Sputum	Illumina	V1-V3	–	18	6
Dang et al. (15)	Airway	Case-control	Sputum	NovaSeq-PE250	V3-V4	19	51	–
Su et al. (16)	Airway	Case-control	Sputum	Illumina	V3-V4	10	23	28
Zhu et al. (17)	Airway	Case-control	Sputum	Illumina	V3-V4	36	–	34
Millares et al. (18)	Airway	Cross-sectional	Sputum	454 pyrosequencing	V1-V3	-	8	8
Feigelman et al. (19)	Airway	Case-control	Sputum	Illumina	NR	4	4	–
Erb-Downward et al. (20)	Airway	Case-control	BALF	454 pyrosequencing	V1-V3	10	4	–
Pragman et al. (21)	Airway	Case-control	BALF	454 pyrosequencing	V3	10	22	–
Einarsson et al. (22)	Airway	Case-control	BALF	Illumina	V1-V2	11	18	–
Mika et al. (23)	Airway	Cross-sectional	BALF	454 pyrosequencing	V3-V5	10	32	–
Gupta et al. (24)	Airway	Case-control	BALF	Illumina	V3-V4	-	14	13
Kim et al. (25)	Airway	Case-control	lung tissue	454 pyrosequencing	NR	13	13	–
Wu et al. (26)	Intestine	Case-control	Fecal	Illumina	V3-V4	22	29	29
Bowerman et al. (27)	Intestine	Case-control	Fecal	16S rRNA gene sequencing	NR	29	28	–

NR, not reported; n_0_, Healthy; n_1_, SCOPD; n_2_, AECOPD; “-”, none.

When characterizing the microbial attributes of different cavities of the host body, including the oral cavity, airway, and intestine, researchers predominantly employed Alpha diversity indices. Analysis of aggregated data revealed significant variations in Alpha diversity indices across different study populations. However, notable heterogeneity existed within studies conducted on the same population with regarded to Alpha diversity indices, which could be attributed to factors such as sample size, types of samples collected, and sampling procedures. (**Supplementary Tables 3**–**5**).

### 3.2 Quality of included studies

Quality was assessed using the NOS. After assessment, a total of two high-quality studies, sixteen medium-quality studies, and two low-quality studies were incorporated into this article. The specific scores obtained for each study based on the NOS are presented in **Figure 1**.

### 3.3 Comparisons of alpha-diversity among HC, SCOPD, and AECOPD

**Figure 3:**
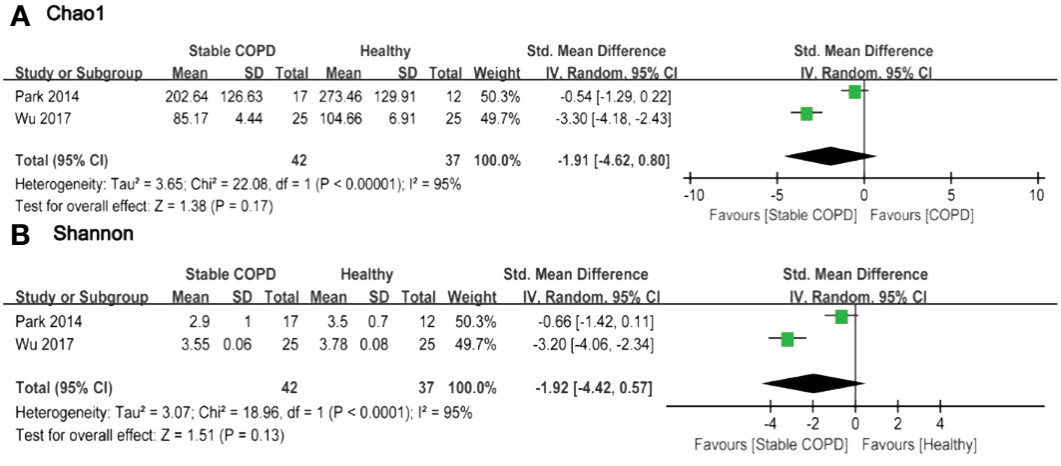
Forest plot of randomized controlled trials comparing the oral microbial alpha-diversity between HC and SCOPD. **(A)** Chao 1 index; **(B)** Shannon index.

**Figure 4:**
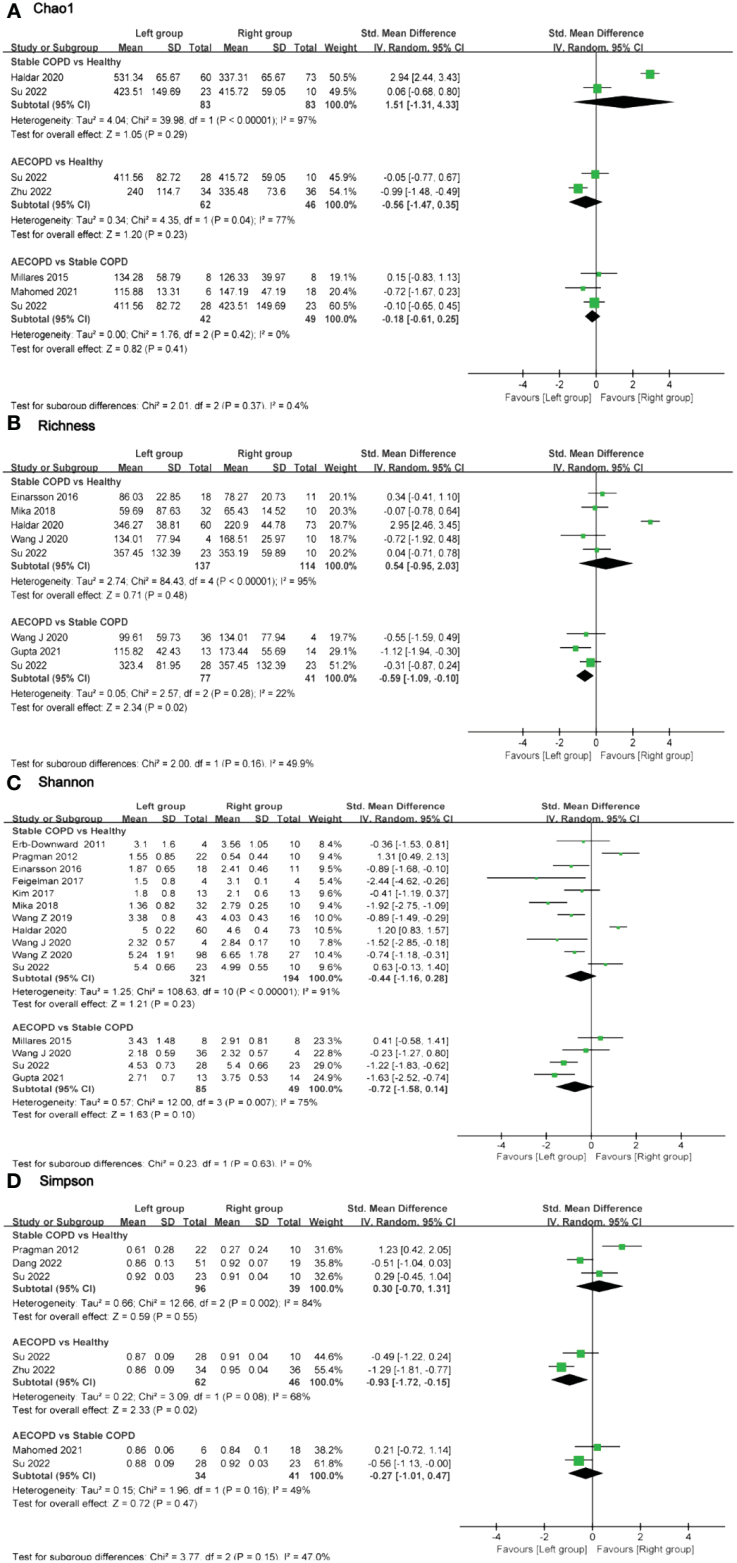
Forest plot of randomized controlled trials comparing the airway microbial alpha-diversity among HC, SCOPD and AECOPD. **(A)** Chao 1 index; **(B)** Richness; **(C)** Shannon index; **(D)** Simpson index.

A total of twenty studies, including 337 HC, 511 SCOPD, and 154 AECOPD, were included in this meta-analysis. In this study, observed species richness and Chao1 index were selected to evaluate microbial richness, while the Shannon index and Simpson index were used to assess microbial evenness. **Figures 3**, **4** present the standardized mean differences and their confidence intervals between the control group and observation group. Among these studies, two focused on oral microbiota using the Chao1 index and Shannon index as indicators of sample alpha-diversity (8, 9). For respiratory microbiota analysis, out of sixteen studies included in this meta-analysis, five reported the Chao1 index, six reported the Richness, thirteen reported the Shannon index, and six reported the Simpson index. Only one out of two studies investigating intestinal microbiota provided data on HC and SCOPD for the Chao1 index, Richness, Shannon index, and Simpson index (26). Additionally, in our search results we found one study reporting both Chao1 index and Shannon index for different severities of SCOPD.

Based on the forest plot, it was evident that a majority of studies indicated lower Alpha-diversity indices in the observation group compared to the control group. Only a few studies reported higher Alpha-diversity indices in the intervention group (11, 18, 21). However, there were no statistically significant differences in Alpha-diversity among most comparison groups. Two studies provided Chao1 and Shannon indices for oral microbiota in 42 SCOPD and 37 HC. The pooled estimate of Chao1 index exhibited substantial heterogeneity (*I* ^2 ^= 95%, *p*<0.0001), with no significant difference observed between groups (SMD=-1.91, 95%CI -4.62 to 0.80) (**Figure 3A**). Similarly, the pooled estimate of Shannon index also demonstrated high heterogeneity (*I* ^2 ^= 95%, *p*<0.0001), with no significant difference found between groups (SMD=-1.92, 95%CI -4.42 to 0.57) (**Figure 3B**).

In terms of airway microbiota diversity, there were no significant differences observed in the comparisons of the Chao1 index and Shannon index among the groups (**Figures 4A, C**). However, regarding the Simpson index, data from two studies including 62 AECOPD patients and 46 HC individuals showed high heterogeneity (*I^2 ^= *68%, *p* = 0.02), with significant differences between groups (SMD = -0.93, 95% CI -1.72 to -0.15). On the other hand, no significant differences were found in the comparisons of the Simpson index for other groups (**Figure 4D**). Three studies provided Richness data for a total of 77 AECOPD patients and 41 SCOPD patients without observing any significant heterogeneity in the pooled analysis (*I^2 ^= *22%, *p* = 0.28), and there were significant differences in terms of Richness (SMD = -0.59, 95% CI -1.09 to -0.10). Whereas no significant difference was found in Richness when comparing SCOPD and HC individuals **Figure 4B**).

Two studies were identified that characterized the diversity of intestinal microbiota between SCOPD and HC. Bowerman et al. observed disparities in intestine microbial composition between SCOPD and HC; however, specific data on diversity indices were not provided in the **Supplementary Materials**, impeding our ability to obtain precise diversity indices. Nevertheless, this study reported no significant differences in diversity levels between SCOPD and HC (*p*Shannon = 0.329, *p*SimpsonInverse = 0.291) (27). Conversely, Wu et al. revealed lower alpha-diversity indices for both SCOPD and AECOPD when compared to HC; AECOPD exhibited even lower diversity than SCOPD (26). Furthermore, Chiu et al., through further investigation, compared Chao1 and Shannon indices of intestinal microbiota among mild, moderate, and severe COPD patients. They discovered a gradual decrease in the Chao1 index with increasing disease severity while observing that the Shannon index was lowest among mild COPD patients and highest among moderate COPD patients (28) (**Supplementary Table 5**).

### 3.4 Comparisons of beta-diversity among HC, SCOPD, and AECOPD

In all the included articles, 16 studies reported on β-diversity, with the most commonly measured being PCoA analysis based on weighted UniFrac distance and Bray-Curtis dissimilarity. **Table 2** reveals that regarding oral microbiota, one study supported differences in microbial composition between SCOPD and HC (8), while another study found no significant differences (9). Concerning airway microbiota, four studies supported differences in microbial structure between SCOPD and HC (11–13, 15), while one study found no significant differences (16); three studies supported differences in microbial composition between AECOPD and HC (12, 16, 17); two studies supported differences in microbial composition between AECOPD and SCOPD (12, 16), while three studies found no significant differences (14, 18, 24). Regarding intestinal microbiota, two studies supported differences in microbial composition between COPD and HC (26, 27).

**Table 2:** Summary of beta diversity assessments in the included studies.

Study	β diversity	Findings	Statistic value
Park et al. (8)	PCoA of Fast UniFrac distances	A clear difference in oral microbial composition between SCOPD and HC.	NR
Wu et al. (9)	PCoA of Weighted UniFrac distances	No significant difference in oral microbial composition between SCOPD and HC.	NR
Wang et al. (10)	NR	NR	NR
Haldar et al. (11)	PCoA of Weighted UniFrac distances	A significant difference in airway microbial composition between SCOPD and HC.	p = 0.01
Wang et al. (12)	PCoA based on Bray-Curtis dissimilarity	A significant difference in airway microbial composition among AECOPD, SCOPD and HC.	p < 0.05
Wang et al. (13)	PCoA of Weighted UniFrac distances	A significant difference in airway microbial composition between SCOPD and HC.	p = 0.004
Mahomed et al. (14)	PCoA of Weighted UniFrac distances	No significant difference in airway microbial composition between SCOPD and AECOPD.	NR
Dang et al. (15)	PCoA analysis based on amplicon sequence variants (ASVs) features distribution Beta diversity analysis based on the Jaccard distance	A clear difference in airway microbial composition between SCOPD and HC. A significant difference in airway microbial composition between SCOPD and HC.	NR p < 0.001
Su et al. (16)	PCoA based on Bray-Curtis dissimilarity	No significant difference in airway microbial composition between SCOPD and HC. A significant difference in airway microbial composition between AECOPD and HC. A significant difference in airway microbial composition between SCOPD and AECOPD.	p = 0.066 p = 0.035 p = 0.001
Zhu et al. (17)	PCoA of Weighted UniFrac distances	A significant difference in airway microbial composition between AECOPD and HC.	p = 0.001
Millares et al. (18)	PCoA based on Bray-Curtis dissimilarity	No significant difference in airway microbial composition between SCOPD and AECOPD.	p = 0.955
Feigelman et al. (19)	NR	NR	NR
Erb-Downward et al. (20)	Principal Components Analysis	NR	NR
Pragman et al. (21)	PCoA of Fast UniFrac distances	A clustering of COPD and HC samples in airway microbial composition.	NR
Einarsson et al. (22)	NR	NR	NR
Mika et al. (23)	NR	NR	NR
Gupta et al. (24)	PCA based on Bray-Curtis dissimilarity	No significant difference in airway microbial composition between SCOPD and AECOPD.	NR
Kim et al. (25)	Principal Components Analysis	NR	NR
Wu et al. (26)	PCoA of Unweighted UniFrac distances	A significant difference in intestine microbial composition among AECOPD, SCOPD and HC.	p < 0.001
Bowerman et al. (27)	PERMANOVA of Bray–Curtis distances	A significant difference in intestine microbial composition between SCOPD and HC.	p < 0.0001

HC, Healthy control; SCOPD, Stable COPD; AECOPD, Acute exacerbations of COPD; NR, Not reported.

### 3.5 Differences in microbial taxa abundance

**Figure 5:**
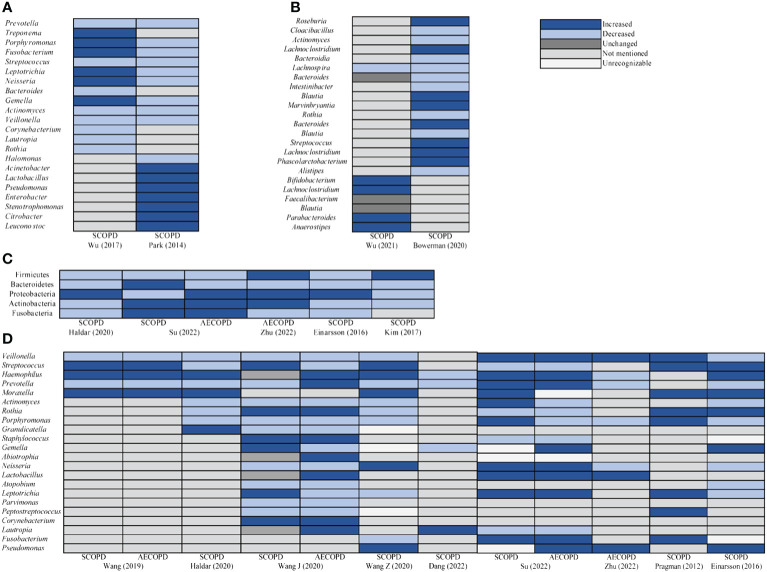
Taxa relative abundance changes in microbiota between SCOPD and AECOPD compared to HC. **(A)** The oral genus comparison of taxa relative abundance between HC and SCOPD. **(B)** The intestinal genus comparison of taxa relative abundance between HC and SCOPD. **(C)** The airway phyla comparison of taxa relative abundance between SCOPD and AECOPD compared to HC. **(D)** The airway genus comparison of taxa relative abundance between SCOPD and AECOPD compared to HC.

Most studies had presented graphical representations of the relative abundance of microbial phyla and genera in both the experimental and control groups. We observed significant alterations in microbial communities depicted in these images, and synthesized these research findings (**Figure 5**). Compared to HC, *Prevotella*, *Streptococcus*, *Actinomyces*, and *Veillonella* of oral microbiota in SCOPD were reduced at the genus level. Most studies supported that Firmicutes, Bacteroidetes, Actinobacteria, and Fusobacteria of airway microbiota at phyla level in SCOPD were decreased. *Haemophilus*, *Lactobacillus*, and *Pseudomonas* were increased, but *Veillonella*, *Prevotella*, *Actinomyces*, *Porphyromonas*, and *Atopobium* were decreased at the genus level in SCOPD. However, the abundance of Bacteroidetes phylum decreased, while the Proteobacteria and Actinobacteria phyla exhibited an increase in the airway microbiota of AECOPD compared to HC. And the abundance of *Haemophilus*, *Lactobacillus* and *Pseudomonas* genera exhibited an increase, whereas *Actinomyces* and *Porphyromonas* showed a decrease. We also observed that *Lachnospira* of intestinal microbiota in SCOPD was reduced at the genus level.

### 3.6 Risk assessment of bias in meta-analysis

The heterogeneity assessment results did not meet our expectations. The forest plot of this meta-analysis revealed high heterogeneity in all summary results, except for the comparison between AECOPD and SCOPD which showed low heterogeneity in Chao1 index, Simpson index, and Richness index. Among the 20 included studies on airway microbiota, Shannon index was the most frequently reported alpha-diversity index with 11 studies reporting differences between SCOPD and HC. Additionally, 5 studies reported differences in Richness index between SCOPD and HC. Sensitivity analysis was conducted to determine sources of heterogeneity for these 11 and 5 studies by sequentially removing each study from the analysis to observe result stability and accuracy. Results remained stable after removing each study one by one. (**Supplementary Figures 1A, B**).

To further validate the reliability of the Shannon index results for airway microbiota, we conducted Begg’s test and Egger’s test to evaluate the potential presence of publication bias. The outcomes revealed that all *p*-values associated with these indicators exceeded 0.05, indicating a lack of evidence supporting publication bias and providing additional support for the robustness of the conclusions derived from our meta-analysis. (**Supplementary Figures 1C, D**)

## 4 Discussion

This study presents the first comprehensive meta-analysis comparing microbial diversity in three different host sites (oral, airway, and intestine) between COPD and HC. The majority of findings suggested a tendency towards reduced microbial diversity during disease states or exacerbations. However, when considering the pooled data, there was no significant decrease in microbial diversity observed between stable and exacerbated COPD patients compared to HC. Notably, two distinct differences were identified in the results: the Simpson index of airway microbiota was lower in AECOPD compared to HC, while Richness was lower in stable COPD (**Figures 4B, D**). These findings indicated a potential reduction in richness and homogeneity of airway microbiota during acute exacerbation. Furthermore, our analysis included two eligible studies on COPD intestinal microbiota; however, one study did not provide sufficient data for comparison using visualization forest plots. Bowerman et al.’s study revealed no significant difference in β-diversity between COPD patients who smoke and those who did not. Furthermore, there were no notable disparities in the microbial composition between patients receiving inhaled corticosteroids, β-agonists, or anticholinergic drugs compared to those who did not receive such medications. AECOPD can be triggered by viral and bacterial infections as well as environmental pollution, among other factors. Pathogens disrupt the dynamic equilibrium of the host’s normal bacterial community, leading to acute deterioration of the disease (29). The forest plot analysis indicated that most AECOPD microbiota exhibiting a decreasing trend in alpha-diversity compared to other groups, which was consistent with the result of Avalos-Fernandez et al.’s meta-analysis; however, overall results did not demonstrate significant differences, this might be constrained by the limited number of included studies (30). Considering the limited number of studies we have included, particularly the scarcity of research on the diversity of oral and intestinal microbiota in COPD, caution should be exercised when interpreting the results from forest plots.

The definition of microbial health clearly emphasizes that health is not a static state but rather a dynamic equilibrium. The characterization of ecological imbalance in microbial communities remains challenging, yet it can be perceived as a disturbance deviating from the original balanced ecological environment (31). While alpha-diversity is often considered crucial for the success or failure of microbial communities, it would be oversimplistic to categorize high-diversity communities as inherently ‘superior’ or more valuable than low-diversity communities to some extent (32). Despite observing higher heterogeneity in our included studies, sensitivity analysis confirmed consistent results regarding the Shannon index of airway microbiota in SCOPD and HC. This comparison encompassed 11 studies and when combined with other forest plot findings comparing both groups, it did not preclude the possibility that there were no significant differences in microbial composition between SCOPD and HC, suggesting an absence of distinct biomarkers between them. Beta-diversity serves as an indicator assessing similarities in microbial community composition among different sample groups by focusing on variations in microbial community structure across samples (33). In this meta-analysis, PCA and PCoA were employed by included studies to discern disparities in microbial community composition among samples. A total of 16 articles reported results from beta-diversity analyses, with half supporting dissimilarities in microbial structure when comparing SCOPD, AECOPD, and HC pairwise (**Table 2**).

Furthermore, a comprehensive analysis of the relative abundance of microbiota in diverse populations with varying conditions was conducted by synthesizing available visual data (**Figure 5**). Notably, sputum sample studies had revealed that more than half of COPD patients harbored potential pathogens, indicating a distinctive microbial composition in their lungs (34). In respiratory diseases, infections primarily contribute to acute exacerbations, with bacterial infections accounting for up to 50% of these cases (35, 36). In COPD specifically, the presence of respiratory bacteria is referred to as colonization and should not be considered benign; instead, it is associated with airway inflammation, aggravated symptoms, and an elevated risk of exacerbation (37). The majority of research findings indicated an elevated presence of *Streptococcus* and *Haemophilus* in the airway microbiota of SCOPD and AECOPD, compared to HC, while a reduction in the abundance of *Streptococcus* was evident in the oral microbiota of SCOPD. *Streptococcus* colonizes the nasopharynx of the human body and can produce various virulence factors, including polysaccharide capsules, which contribute to the development of diseases such as pneumonia and meningitis (38). *Haemophilus* also resides in the nasal pharynx of humans, with the majority isolated from respiratory tracts being untyped *Haemophilus*. Although they rarely cause invasive diseases, COPD leads to significant structural and functional changes in airways that compromise the host’s immune response to respiratory pathogens. This creates favorable conditions for *Haemophilus* to establish persistent infections, with approximately half of all isolates in COPD patients belonging to *Haemophilus* (37, 39, 40). Previous studies had demonstrated an elevation in the total bacterial load among individuals with COPD, which was associated with compromised respiratory health. In comparison to HC, sputum samples from COPD patients exhibited a higher prevalence of *Haemophilus* and *Streptococcus*, and their presence positively correlated with levels of neutrophil elastase and IL-1β in the sputum (41), consistent with our microbial taxa relative abundance findings. Furthermore, a reduction in the relative abundance of *Veillonella*, *Prevotella*, *Actinomyces*, and *Porphyromonas* was observed in COPD. *Veillonella* is a gram-negative anaerobic bacterium that belongs to normal oral commensals; however, its precise role remains elusive (42). Einarsson et al. reported greater quantities of *Veillonella* in HC compared to COPD. It had been noted that the abundance of *Prevotella* decreased during asthma and COPD while pathogenic bacteria proliferated instead; nevertheless, the potential steady-state function of *Prevotella* within healthy lungs largely remains unknown (43). Our summarized findings lent support to the notion that *Veillonella*, *Prevotella*, *Actinomyces*, and *Porphyromonas* might confer beneficial effects both during states of health or disease.

Smoking is closely associated with COPD and contributes to an imbalance in oral microbiota. Jia et al. discovered that smokers have a higher abundance of *Moraxella* and *Rothia* in their oral cavity (44). *Rothia*, a Gram-positive bacterium, naturally resides in the human oral cavity. In patients with bronchiectasis, the presence of *Rothia* was found to be negatively correlated with pro-inflammatory factors such as IL-8, IL-1β, MMP-1, MMP-8, and MMP-9 in sputum (45). Our findings also indicated that the quantity of *Moraxella* and *Rothia* in airway microbiota were linked to the overall health status of the host. This might be attributed to the body’s autonomous regulation of *Rothia* during disease states to inhibit activation of NF-kB pathway and subsequently suppress the release of various inflammatory factors (46). Melo-Dias et al.’s study further supported this notion by demonstrating a negative correlation between abundance of *Rothia* and multiple inflammatory factors (47).

Our meta-analysis compared the microbial diversity between different disease states of COPD and healthy HC. A total of 20 studies involving 1002 participants were included in our analysis. We did not find any significant difference in the alpha-diversity of the microbiota between COPD and HC; however, we observed an increasing trend of certain microbiota in the diseased state. Nevertheless, there are several limitations to our study: (1) The number of studies investigating oral and intestinal microbiota was limited, with relatively small sample sizes. Therefore, the evidence obtained from our meta-analysis may be insufficient. Future research should aim to increase the number of studies on oral and intestinal microbiota and validate our findings in larger populations. (2) Most of the included studies relied on 16S rRNA gene sequencing, which restricts interpretation at a species level. Additionally, few studies provided sequencing datasets, making it challenging for researchers to integrate data for analysis. (3) Our forest plot results exhibited high heterogeneity; however, sensitivity analyses yielded consistent outcomes. (4) The estimation of M and SD using median and interquartile range based on figures provided by literature might introduce some errors into alpha-diversity indices.

## 5 Conclusion

The majority of published research findings supported the hypothesis that COPD exhibit lower alpha diversity compared to HC. However, our meta-analysis did not confirm this observation; but most studies did report significant microbial difference in beta diversity between COPD and HC. Furthermore, changes in the relative abundance of microbial communities in different cavities of the body (oral, airway and intestine) may occur, based on variations in the disease status of patients. However, other studies with the same relevance are needed to further investigate the characteristics and mechanisms of microbial involvement in the oral-airway-intestine axis in patients with COPD. And Future studies should be conducted on a larger scale with more rigorous methods.

## Data availability statement

The original contributions presented in the study are included in the article/**Supplementary Material**. Further inquiries can be directed to the corresponding authors.

## Author contributions

WH: Writing – review & editing. ZK: Writing – original draft, Writing – review & editing. KL: Writing – original draft. ZQ: Writing – original draft. YW: Writing – original draft. YML: Writing – original draft. YNL: Writing – original draft. XY: Writing – review & editing.
