# Supplementary material for: The alterations of oral, airway and intestine microbiota in chronic obstructive pulmonary disease: a systematic review and meta-analysis
Source: Front Immunol. 2024 May 8;15:1407439. doi: 10.3389/fimmu.2024.1407439 (PMC11109405; doi:10.3389/fimmu.2024.1407439)
Supplement: Supplementary file 1 [file DataSheet_1.docx]

Supplementary Material

**Supplementary Table 1.** Systematic search detail.

**Supplementary Table 2.** Quality assessment of the included studies using the Newcastle-Ottawa Scale.

**Supplementary Table 3.** Alpha-diversity index of oral microbiota between HC and SCOPD.

**Supplementary Table 4.** Alpha-diversity index of airway microbiota among HC, SCOPD and AECOPD.

**Supplementary Table 5.** Alpha-diversity index of intestinal microbiota in HC and COPD.

**Supplementary Figure 1.** Sensitivity analysis assessing heterogeneity and publication bias.

**Supplementary Table 1. Systematic search detail.**

- 1. **Pubmed search stratergy for 1005**

| **Search** | **Hits** |
| --- | --- |
| 1#  (((((((((((((((((((((("Microbiota"[Mesh]) OR (Microbiota[Title/Abstract])) OR (Microbiotas[Title/Abstract])) OR (Microbial Community[Title/Abstract])) OR (Community, Microbial[Title/Abstract])) OR (Microbial Communities[Title/Abstract])) OR (Microbial Community Composition[Title/Abstract])) OR (Community Composition, Microbial[Title/Abstract])) OR (Composition, Microbial Community[Title/Abstract])) OR (Microbial Community Compositions[Title/Abstract])) OR (Microbial Community Structure[Title/Abstract])) OR (Community Structure, Microbial[Title/Abstract])) OR (Microbial Community Structures[Title/Abstract])) OR (Microbiome[Title/Abstract])) OR (Microbiomes[Title/Abstract])) OR (Human Microbiome[Title/Abstract])) OR (Human Microbiomes[Title/Abstract])) OR (Microbiome, Human[Title/Abstract])) OR (Flora[Title/Abstract])) OR (Microflora[Title/Abstract])) OR (Dysbiosis[Title/Abstract])) OR (Ecosystem[Title/Abstract])) OR (Bacteria[Title/Abstract]) | 668909 |
| 2#  **(**(((((((((("Pulmonary Disease, Chronic Obstructive"[Mesh]) OR (Pulmonary Disease, Chronic Obstructive[Title/Abstract])) OR (Chronic Obstructive Lung Disease[Title/Abstract])) OR (Chronic Obstructive Pulmonary Diseases[Title/Abstract])) OR (COAD[Title/Abstract])) OR (COPD[Title/Abstract])) OR (Chronic Obstructive Airway Disease[Title/Abstract])) OR (Chronic Obstructive Pulmonary Disease[Title/Abstract])) OR (Airflow Obstruction, Chronic[Title/Abstract])) OR (Airflow Obstructions, Chronic[Title/Abstract])) OR (Chronic Airflow Obstructions[Title/Abstract])) OR (Chronic Airflow Obstruction[Title/Abstract]) | 104615 |
| **3#：1# AND 2#**  ((((((((((((((((((((((("Microbiota"[Mesh]) OR (Microbiota[Title/Abstract])) OR (Microbiotas[Title/Abstract])) OR (Microbial Community[Title/Abstract])) OR (Community, Microbial[Title/Abstract])) OR (Microbial Communities[Title/Abstract])) OR (Microbial Community Composition[Title/Abstract])) OR (Community Composition, Microbial[Title/Abstract])) OR (Composition, Microbial Community[Title/Abstract])) OR (Microbial Community Compositions[Title/Abstract])) OR (Microbial Community Structure[Title/Abstract])) OR (Community Structure, Microbial[Title/Abstract])) OR (Microbial Community Structures[Title/Abstract])) OR (Microbiome[Title/Abstract])) OR (Microbiomes[Title/Abstract])) OR (Human Microbiome[Title/Abstract])) OR (Human Microbiomes[Title/Abstract])) OR (Microbiome, Human[Title/Abstract])) OR (Flora[Title/Abstract])) OR (Microflora[Title/Abstract])) OR (Dysbiosis[Title/Abstract])) OR (Ecosystem[Title/Abstract])) OR (Bacteria[Title/Abstract])) AND (((((((((((("Pulmonary Disease, Chronic Obstructive"[Mesh]) OR (Pulmonary Disease, Chronic Obstructive[Title/Abstract])) OR (Chronic Obstructive Lung Disease[Title/Abstract])) OR (Chronic Obstructive Pulmonary Diseases[Title/Abstract])) OR (COAD[Title/Abstract])) OR (COPD[Title/Abstract])) OR (Chronic Obstructive Airway Disease[Title/Abstract])) OR (Chronic Obstructive Pulmonary Disease[Title/Abstract])) OR (Airflow Obstruction, Chronic[Title/Abstract])) OR (Airflow Obstructions, Chronic[Title/Abstract])) OR (Chronic Airflow Obstructions[Title/Abstract])) OR (Chronic Airflow Obstruction[Title/Abstract])) | 1576 |
| Limits：English | 1451 |
| Limits：Humans | 1123 |
| Limits：2003-current | **1005** |

- 1. **Web of Science search stratergy for 1589**

| **Search** | **Hits** |
| --- | --- |
| (TS=(Microbiota) OR AB=(Microbiota OR Microbiotas OR Microbial Community OR Community, Microbial OR Microbial Communities OR Microbial Community Composition OR Community Composition, Microbial OR Composition, Microbial Community OR Microbial Community Compositions OR Microbial Community Structure OR Community Structure, Microbial OR Microbial Community Structures OR Microbiome OR Microbiomes OR Human Microbiome OR Human Microbiomes OR Microbiome, Human OR Flora OR Microflora OR Dysbiosis OR Ecosystem OR Bacteria)) AND (TS=(Pulmonary Disease, Chronic Obstructive) OR AB=(Pulmonary Disease, Chronic Obstructive OR Chronic Obstructive Lung Disease OR Chronic Obstructive Pulmonary Diseases OR COAD OR COPD OR Chronic Obstructive Airway Disease OR Chronic Obstructive Pulmonary Disease OR Airflow Obstruction, Chronic OR Airflow Obstructions, Chronic OR Chronic Airflow Obstructions OR Chronic Airflow Obstruction)) | 2156 |
| Limits：2003-current | 1800 |
| Limits：English | **1589** |

- 1. **Embase search stratergy for 1867**

| **Search** | **Hits** |
| --- | --- |
| 1#  microbiota:ab,ti OR microbiotas:ab,ti OR 'microbial community':ab,ti OR 'community, microbial':ab,ti OR 'microbial communities':ab,ti OR 'microbial community composition':ab,ti OR 'community composition, microbial':ab,ti OR 'composition, microbial community':ab,ti OR 'microbial community compositions':ab,ti OR 'microbial community structure':ab,ti OR 'community structure, microbial':ab,ti OR 'microbial community structures':ab,ti OR microbiome:ab,ti OR microbiomes:ab,ti OR 'human microbiome':ab,ti OR 'human microbiomes':ab,ti OR 'microbiome, human':ab,ti OR flora:ab,ti OR microflora:ab,ti OR dysbiosis:ab,ti OR ecosystem:ab,ti OR bacteria:ab,ti | 738959 |
| 2#  'pulmonary disease, chronic obstructive':ab,ti OR 'chronic obstructive lung disease':ab,ti OR 'chronic obstructive pulmonary diseases':ab,ti OR coad:ab,ti OR copd:ab,ti OR 'chronic obstructive airway disease':ab,ti OR 'chronic obstructive pulmonary disease':ab,ti OR 'airflow obstruction, chronic':ab,ti OR 'airflow obstructions, chronic':ab,ti OR 'chronic airflow obstructions':ab,ti OR 'chronic airflow obstruction':ab,ti | 141353 |
| 3#  #1 AND #2 | 2398 |
| 4#  #3 AND [english]/lim AND [humans]/lim | 1974 |
| 5#  #4 AND (2003:py OR 2004:py OR 2005:py OR 2006:py OR 2007:py OR 2008:py OR 2009:py OR 2010:py OR 2011:py OR 2012:py OR 2013:py OR 2014:py OR 2015:py OR 2016:py OR 2017:py OR 2018:py OR 2019:py OR 2020:py OR 2021:py OR 2022:py OR 2023:py) | **1867** |

- 1. **Cochrane search stratergy for 254**

| **Search** | **Hits** |
| --- | --- |
| #1 MeSH descriptor: [Microbiota] explode all trees | 1741 |
| #2 (Microbiota or Microbiotas or Microbial Community or Community, Microbial or Microbial Communities or Microbial Community Composition or Community Composition, Microbial or Composition, Microbial Community or Microbial Community Compositions or Microbial Community Structure or Community Structure, Microbial or Microbial Community Structures or Microbiome or Microbiomes or Human Microbiome or Human Microbiomes or Microbiome, Human or Flora or Microflora or Dysbiosis or Ecosystem or Bacteria):ti,ab,kw (Word variations have been searched) | 26238 |
| #3 #1 or #2 | 26241 |
| #4 MeSH descriptor: [Pulmonary Disease, Chronic Obstructive] explode all trees | 7219 |
| #5 (Pulmonary Disease, Chronic Obstructive or Chronic Obstructive Lung Disease or Chronic Obstructive Pulmonary Diseases or COAD or COPD or Chronic Obstructive Airway Disease or Chronic Obstructive Pulmonary Disease or Airflow Obstruction, Chronic or Airflow Obstructions, Chronic or Chronic Airflow Obstructions or Chronic Airflow Obstruction):ti,ab,kw (Word variations have been searched) | 24513 |
| #6 #4 or #5 | 24834 |
| #7 #3 and #6 with Cochrane Library publication date Between May 2003 and May 2023 | **261** |
| Cochrane Reviews | 7 |
| Trials | **254** |

**Supplementary Table 2.** **Quality assessment of the included studies using the Newcastle-Ottawa Scale.**

| **Study** | **Selection** | **Comparability** | **Exposure** | **Total** |
| --- | --- | --- | --- | --- |
| [HeeKuk Park](https://pubmed.ncbi.nlm.nih.gov/?term=Park+H&cauthor_id=25329665),2014 | 1 | 0 | 2 | 3 |
| [Xingwen Wu](https://pubmed.ncbi.nlm.nih.gov/?term=Wu+X&cauthor_id=28748030),2017 | 3 | 1 | 2 | 6 |
| [Zhang Wang](https://pubmed.ncbi.nlm.nih.gov/?term=Wang+Z&cauthor_id=31170986),2019 | 2.5 | 1.5 | 2 | 6 |
| [Koirobi Haldar](https://pubmed.ncbi.nlm.nih.gov/?term=Haldar+K&cauthor_id=32664956),2020 | 3 | 1 | 2 | 6 |
| [Juan Wang](https://pubmed.ncbi.nlm.nih.gov/?term=Wang+J&cauthor_id=32811432),2020 | 3 | 0.5 | 2 | 5.5 |
| [Zhang Wang](https://pubmed.ncbi.nlm.nih.gov/?term=Wang+Z&cauthor_id=32849386),2020 | 3 | 1 | 2 | 6 |
| [T Goolam Mahomed](https://pubmed.ncbi.nlm.nih.gov/?term=Goolam+Mahomed+T&cauthor_id=34611216),2021 | 2.5 | 1.5 | 2 | 6 |
| [Xiaomin Dang](https://pubmed.ncbi.nlm.nih.gov/?term=Dang+X&cauthor_id=36532417),2022 | 3 | 1 | 2 | 6 |
| [Linfan Su](https://pubmed.ncbi.nlm.nih.gov/?term=Su+L&cauthor_id=35123490),2022 | 3 | 2 | 2 | 7 |
| [Shu-Fen Zhu](https://pubmed.ncbi.nlm.nih.gov/?term=Zhu+SF&cauthor_id=36314008),2022 | 3 | 1.5 | 2 | 6.5 |
| [Laura Millares](https://pubmed.ncbi.nlm.nih.gov/?term=Millares+L&cauthor_id=26632844),2015 | 1 | 1 | 2 | 4 |
| [Rounak Feigelman](https://pubmed.ncbi.nlm.nih.gov/?term=Feigelman+R&cauthor_id=28187782),2017 | 2 | 0 | 2 | 4 |
| [John R Erb-Downward](https://pubmed.ncbi.nlm.nih.gov/?term=Erb-Downward+JR&cauthor_id=21364979),2011 | 2 | 0 | 2 | 4 |
| [Alexa A Pragman](https://pubmed.ncbi.nlm.nih.gov/?term=Pragman+AA&cauthor_id=31174538),2019 | 1 | 0 | 2 | 3 |
| [G G Einarsson](https://pubmed.ncbi.nlm.nih.gov/?term=Einarsson+GG&cauthor_id=27146202),2016 | 3 | 1.5 | 2 | 6.5 |
| [Moana Mika](https://pubmed.ncbi.nlm.nih.gov/?term=Mika+M&cauthor_id=29992131),2018 | 2.5 | 1 | 2 | 5.5 |
| [Shashank Gupta](https://pubmed.ncbi.nlm.nih.gov/?term=Gupta+S&cauthor_id=33597669),2021 | 2 | 1 | 2 | 5 |
| [Hyun Jung Kim](https://pubmed.ncbi.nlm.nih.gov/?term=Kim+HJ&cauthor_id=28408748),2017 | 3 | 1 | 2 | 6 |
| [Yonghong Wu](https://pubmed.ncbi.nlm.nih.gov/?term=Wu+Y&cauthor_id=33181175),2021 | 2 | 1 | 2 | 5 |
| [Kate L Bowerman](https://pubmed.ncbi.nlm.nih.gov/?term=Bowerman+KL&cauthor_id=33208745),2020 | 3 | 2 | 2 | 7 |

**Supplementary Table 3.** Alpha-diversity index of oral microbiota between HC and SCOPD.

**Supplementary Table 4.** Alpha-diversity index of airway microbiota among HC, SCOPD and AECOPD.

**Supplementary Table 5.** Alpha-diversity index of intestinal microbiota in HC and COPD.

The aforementioned three tables can be located in the supplementary Excel spreadsheet.

**
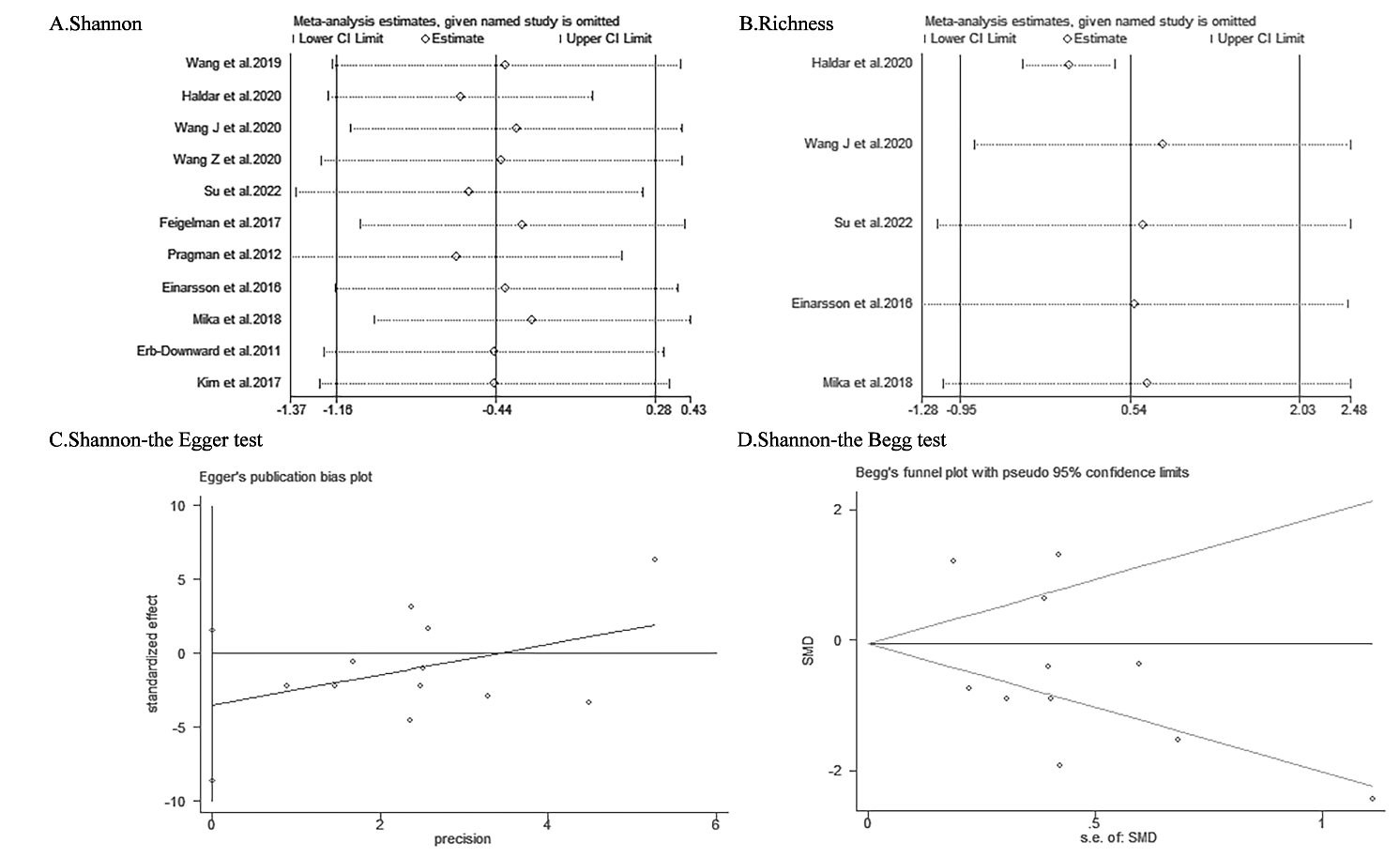
**

**Supplementary Figure 1.** **Sensitivity analysis assessing heterogeneity and publication bias. A. Shannon, B. Richness, C. Shannon-the Egger test, D. Shannon-the Begg test**
